# Gerontology at the Intersection of Religion and Families: Honoring the Legacy of Vern Bengtson

**DOI:** 10.1093/geroni/igaa057.2265

**Published:** 2020-12-16

**Authors:** Alex Bishop, Merril Silverstein, Monika Ardelt

## Abstract

The topic of religion and spirituality in later life has received intermittent but regular attention in the field of social and behavioral gerontology over the past few decades. To the extent that religion and spirituality have been linked to better health, improved well-being, and harmonious family functioning has renewed interest in this area of scholarly inquiry. Along with these positive outcomes, religion has also been examined as the basis for family conflict, as well in terms of its inverse in the transmission of secularity across generations. This symposium will communicate empirical results, theoretical insights, and conceptual developments inspired by the career contributions of Dr. Vern Bengtson, whose landmark studies have enriched the field of gerontology in the areas of intergenerational solidarity, spirituality in later life, the transmission of religion across generations, and life-course approaches to the study of family relationships. Five eminent scholars whose work touches on these areas will be represented in this symposium. The paper by Linda George compares intergenerational religious socialization and moral development. The paper by Robert Taylor and Linda Chatters examines the role of supportive church and family networks among older African-Americans. The paper by Ellen Idler addresses aging and religious in a context of secularization. The paper by Andy Achenbaum considers spiritual dimensions of friendship and meanings of aging. The paper by Merril Silverstein integrates intergenerational and temporal continuity in religious practice and identity. The discussant Monika Ardelt has contributed important scholarly work in the areas of religion, spirituality, and wisdom in later life. Religion, Spirituality and Aging Interest Group Sponsored Symposium.

